# Critical appraisal of FNAC in the diagnosis of primary papillary carcinoma arising in thyroglossal cyst: A case report with review of the literature on FNAC and its diagnostic pitfalls

**DOI:** 10.4103/0970-9371.66697

**Published:** 2010-01

**Authors:** Kiran Agarwal, Vandana Puri, Smita Singh

**Affiliations:** Department of Pathology, Lady Hardinge Medical College, New Delhi, India

**Keywords:** Papillary carcinoma, thyroglossal cyst, FNAC

## Abstract

The incidence of primary papillary carcinoma arising in thyroglossal cyst is rare and occurs in <1% of thyroglossal cysts. Even rarer is its diagnosis by pre-operative fine needle aspiration cytology (FNAC). Only 15 such cases diagnosed by FNAC have been previously reported in the literature. In this article, cytomorphology of the current case is presented along with a review of the literature on FNAC and its diagnostic pitfalls.

## Introduction

Thyroglossal duct cyst is the most common nonodontogenic cyst of the adult population, presenting as midline neck swellings. The incidence of papillary carcinoma arising in the thyroglossal duct cyst is rare (<1%). Approximately 150 cases of papillary carcinoma arising in the thyroglossal duct cyst have previously been reported in the literature.[1,2] There are very few case reports of these cases diagnosed on fine needle aspiration cytology (FNAC).[1,3–8] Thyroglossal duct cyst carcinomas are usually asymptomatic and not suspected preoperatively in most instances; hence, the need for careful pathological examination of these cysts. In this article, cytological features of the present case are described along with diagnostic pitfalls and review of the literature on FNAC.

## Case Report

A 22-year-old asymptomatic female presented to the surgical outpatient department with a history of midline neck swelling of two years duration. There was no history of prior radiation. On examination, a well-circumscribed, painless, firm, fluctuant, 2 × 2 cm mass in the midline of the neck at the level of the hyoid was found. It showed vertical movement on deglutition. A clinical diagnosis of solitary thyroid nodule was made. There was also right submandibular lymph node enlargement (approx 1.5 cm in diameter). The patient was subsequently referred for FNAC.

## Cytology and Histology

**Figure 1:**
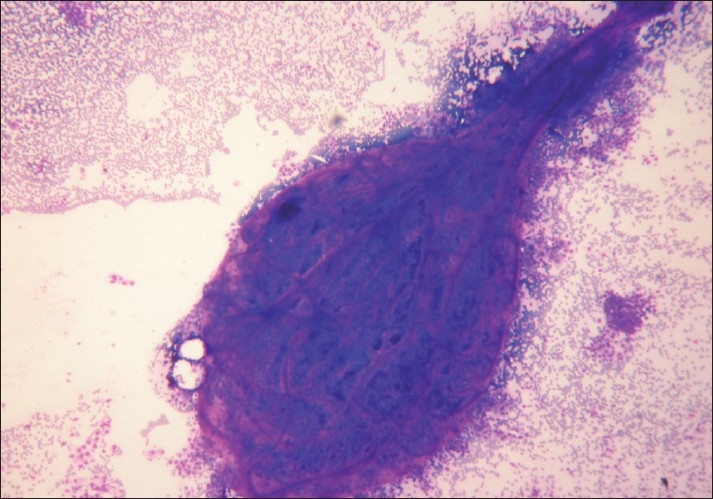
Big papillary clusters having fibrovascular core (Giemsa, ×100)

**Figure 2:**
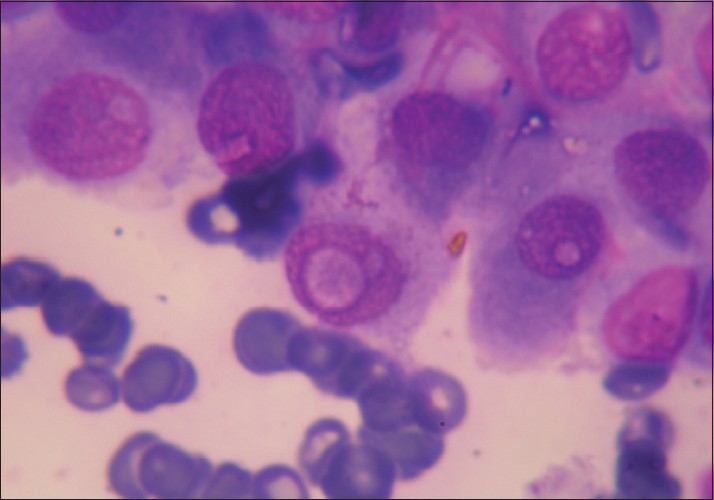
Follicular epithelial cells showing intranuclear cytoplasmic inclusions (Pap, ×1000)

Giemsa and PAP-stained highly cellular FNAC smears were composed of big papillary clusters having a fibrovascular core [Figure 1] and few monolayered sheets of follicular epithelial cells. Few cells showed intranuclear cytoplasmic inclusions [Figure 2] and nuclear grooving in the background of scant colloid and many cyst macrophages. Individual cells were round to cuboidal, with a moderate amount of well-defined cytoplasm and single nuclei. In view of the above findings, diagnosis of papillary carcinoma of the thyroid was suggested. Ultrasonography, computed tomography (CT) scan and biopsy of the thyroid were advised. FNAC smears from the right submandibular lymph node revealed a reactive lymph node. Ultrasound of the neck revealed an unremarkable thyroid and a cystic lesion measuring 2 × 1 cm, separate from the thyroid. On a thyroid scan, both lobes of the thyroid and isthmus showed normal uptake. The cyst did not show any uptake. There were no cold spots in the thyroid. At the time of neck exploration, a small cystic mass measuring 2 × 1 cm and attached to the hyoid bone was found. The thyroid gland was found to be unremarkable.

Hence, preoperative diagnosis of thyroglossal cyst was made and Sistrunk’s operation was performed.

**Figure 3:**
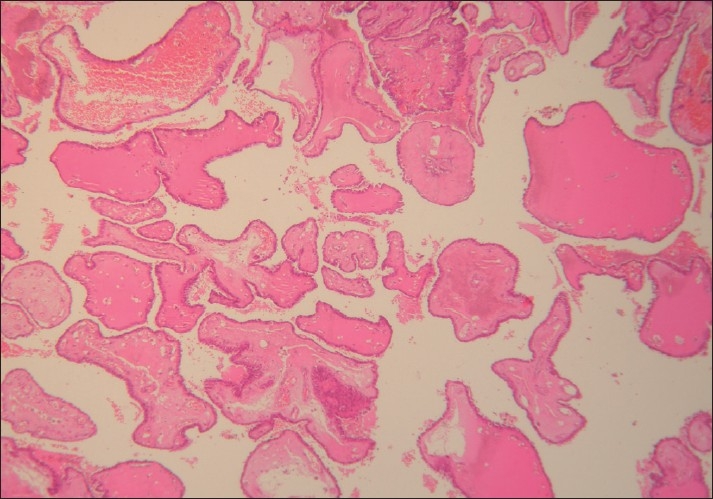
Multiple branching papillae with thin fibro-vascular core (H and E, ×100)

**Figure 4:**
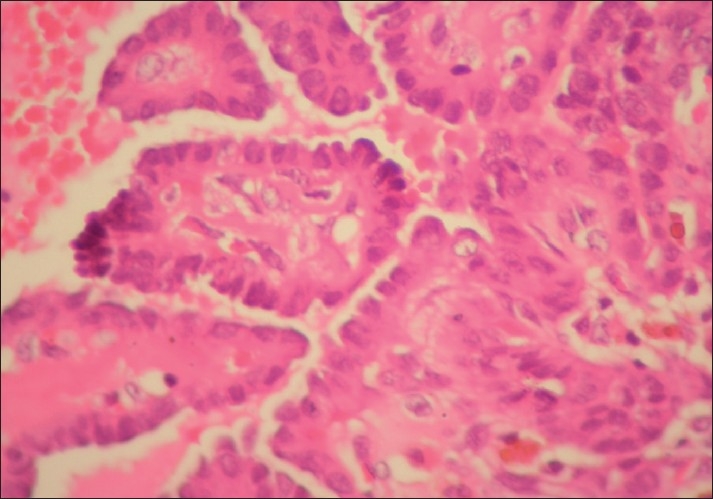
Cells showed ground glass nuclei with few showing intranuclear cytoplasmic inclusions (H and E, ×400)

The cyst was resected and sent for histopathological examination. Gross examination showed a small cystic mass measuring 2 × 1 cm, with an attached part of hyoid bone and skeletal muscle. Cut-section of the cyst showed multiple small friable papillary projections filling the entire cyst. Microscopically, the cyst showed multiple branching papillae with a thin fibrovascular core projecting into its lumen [Figure 3]. The papillae were lined by cuboidal to flattened epithelial cells. The cells showed ground glass nuclei, with few showing intranuclear cytoplasmic inclusions and grooves [Figure 4]. The cyst wall showed infiltration by the tumor. The adjacent thyroid gland was unremarkable. The hyoid bone and skeletal muscle were uninvolved. Hence, the final diagnosis of primary papillary carcinoma of thyroglossal duct cyst was made.

## Discussion

Thyroglossal duct cyst is the most common nonodontogenic cyst, prevalence being 7% of the adult population.[1] Carcinomas arising in the thyroglossal duct cyst are generally of two types: thyrogenic carcinoma and squamous carcinoma. The thyrogenic carcinoma most often arises from thyroembryonic rests in the duct or cyst and the squamous carcinoma arises from the metaplastic columnar epithelium.[9]

Incidence of papillary carcinoma arising in the thyroglossal duct cyst is <1%, and it is usually seen in younger women, with a sex ratio of 1.5:1.[1,9] This patient was a 22-year-old female.

Literature reveals 15 cases of preoperative FNAC of the thyroglossal duct cysts with papillary carcinoma [Table 1]. Nine of the 15 cases were diagnosed as papillary thyroid carcinoma.[1,3–7,10–12] Only four cases were reported as thyroglossal duct cyst papillary carcinoma.[8,13–15] Two case reports were inconclusive.[16,17] Most of the cases on FNAC belonged to the age group of 21–65 years.

**Table 1:** Clinical and cytomorphological features of reported cases

Major findings	Diagnosis	Age	Sex	Source
Papillary cluster	PTC	NG	NG	Kini *et al*.[3]
Papillary fragments	PTC	57	M	Kimberly *et al*.[4]
Papillary formations	PTC	68	M	Katz and Hachigian[10]
Papillary groups, intranuclear inclusions, multinucleated giant cells	PTC	58	F	Pitts *et al*.[5]
Papillary fragments	PTC	21	F	Prasad *et al*.[6]
Papillary formations, psammoma bodies, macrophages	PTC	65	F	Kashkari[7]
Papillary fragments	PTC	52	F	Martin-Perez *et al*.[11]
Papillary elements	PTC	60	M	Kennedy *et al*.[12]
Papillary fragments, nuclear pseudoinclusions, abundant colloid, psammoma bodies, multinucleate giant cells	PTC	30	M	Yang *et al*.[1]
Macrophages, epithelial cells	TDC	42	M	Chen[8]
Abundant colloid, degenerate cells	TDC	43	F	Hilger *et al*.[13]
Markedly atypical epilthelial cells	TDC	29	M	Bardales *et al*.[14]
Abundant colloid, few degenerate cells	TDC	55	M	Vera-Sempere *et al*.[15]
Not described	Benign	42	F	Pacheco-Ojeda *et al*.[16]
Not given	Benign	32	F	Silverman *et al*.[17]

NG: Not given

PTC: Papillary thyroid carcinoma

TDC: Thyroglossal duct cyst

M: Male

F: Female

The cytomorphological features on FNAC have been described by few authors. Some considered diagnosis of papillary thyroid carcinoma on the basis of papillary fragments, while others also described intranuclear pseudoinclusions and multinucleated giant cells.[5–7,12] The diagnosis of thyroglossal duct cyst was made on the basis of macrophages and benign epithelial cells.[8,13,14] None of the case was diagnosed as papillary carcinoma arising in the thyroglossal duct cyst.

Although diagnostic criteria of papillary carcinoma thyroid and thyroglossal duct cyst on FNAC have been defined, diagnostic pitfalls in giving accurate diagnosis of papillary carcinomas arising in the thyroglossal duct cyst are very common. Major criteria are high cellularity, papillary formations, cells with enlarged nuclei showing anisonucleosis and powdery chromatin and definite nucleoli. Intranuclear pseudoinclusions and grooves are significant. Psammoma bodies, multinucleate giant cells and ropy colloid are variably present.[1]

FNAC yielded correct results in only 50–66% of cases.[1,14] The false-negative result on FNAC was due to cystic fluid that was aspirated, leading to hypocellularity from dilution due to cystic contents.[1] Hence, diligent search is a must to diagnose malignant cells. Also, as cystic fluid is often the initial specimen obtained during FNAC of papillary carcinomas and thyroglossal duct cysts, repeat aspirate is important to rule out any residual mass.

Another pitfall in the diagnosis is locating the origin of the tumor by cytological testing alone. In cases of solitary thyroid nodule, entity of the thyroglossal cyst must also be kept in mind and on finding malignancy on FNAC, CT scan or ultrasound should be advised to decide the origin. This can prevent thyroidectomy and its complications.

FNAC also lacks in finding cyst wall infiltration by tumor cells. It is important to quote cyst wall infiltration in the diagnosis as the treatment and prognosis depend on this. If the cancer has not invaded beyond the cyst wall, simple complete excision of the cyst using the Sistrunk’s procedure is adequate.[18] If the cancer has invaded beyond the cystic wall, a total or subtotal thyroidectomy is recommended.[1]

Hence, location of the target lesion, careful searching for malignant cells and repeat FNAC are the key to successful diagnosis. Prognosis of papillary carcinomas is good, whether it occurs in the thyroglossal duct cyst or in the thyroid proper, but frequency of lymph node metastasis is lower in papillary carcinomas arising in the thyroglossal duct cyst than that arising in the thyroid proper.[1]

This case is reported in view of the rarity of pre-operative FNAC diagnosis of this entity and to highlight the diagnostic pitfalls and importance of FNAC to plan a proper surgical management.
